# The lateralization and distalization index is more reliable than angular radiographic measurements in reverse shoulder arthroplasty

**DOI:** 10.1007/s00402-024-05448-6

**Published:** 2024-07-15

**Authors:** Ahmet Emin Okutan, Serkan Surucu, Hubert Laprus, Patric Raiss

**Affiliations:** 1 Department of Orthopaedic Surgery, Samsun University School of Medicine, Samsun, Turkey; 2 Department of Orthopaedics and Rehabilitation, Yale University, New Haven, USA; 3 St Luke’s Hospital, Bielsko-Biala, Poland; 4 Orthopadische Chirurgie Munchen, Munich, Germany

**Keywords:** Distalization, DSA, Lateralization, LSA, Reverse shoulder arthroplasty

## Abstract

**Background:**

The lateralization shoulder angle (LSA) and distalization shoulder angle (DSA) are used to reproducibly measure lateralization and distalization after reverse shoulder arthroplasty (RSA). However, LSA and DSA may not offer a precise measurement of humeral lateralization and distalization and this relationship has not been explored. The aim of this study was to evaluate the validity of these measurements and to propose new measurement methods to estimate implant lateralization and distalization.

**Methods:**

3D models were constructed from computed tomography (CT) scans of 30 patients using a software platform. For each patient 24 different RSA modifications were created, resulting in 720 different RSA configurations. For each configuration LSA and DSA angles as well as lateralization and distalization distances were measured. Moreover, for each configuration two new measurements were done: the lateralization index (LI) and distalization index (DI). Correlations of the lateralization and distalization parameters were evaluated between measurements.

**Results:**

Weak correlations were founded between LSA and lateralization (*r* = 0.36, *p* < 0.01), whereas moderate correlations were observed between LI and lateralization (*r* = 0.72, *p* < 0.01). No significant correlations were found between DSA and distalization (*r* = 0.17, *p* = 0.113). In contrast, moderate correlations were identified between DI and distalization (*r* = 0.69, *p* < 0.01).

**Conclusion:**

LI and DI are more reliable methods to estimate implant lateralization and distalization compared to angular radiographic measurements. However, the prognostic significance in predicting clinical outcomes after RSA remains unknown.

**Supplementary Information:**

The online version contains supplementary material available at 10.1007/s00402-024-05448-6.

## Introduction

Increasing numbers of reverse shoulder arthroplasties (RSAs) are being performed worldwide due to the aging demographics and expanded indications [1]. Moreover, reverse shoulder prosthesis have undergone substantial design changes [2]. The medialized center of rotation concept initially introduced by Grammont was modified over time with more lateralization on the glenoid side and modifications of the neck-shaft angle on the humeral side [3]. Multiple studies have indicated that a distalized and medialized configuration may enhance overhead motion, while a more lateralized configuration is associated with improved axial rotation [4–6]. Therefore, a number of radiographic measurement methods have been described to assess lateralization and distalization [7–9].

One common method to measure lateralization and distalization in RSA are the ‘lateralization shoulder angle’ (LSA) and ‘distalization shoulder angle’ (DSA), which were recently introduced by Boutsiadis et al. [7]. They reported that LSA and DSA significantly correlate with range of motion and functional outcomes after RSA. Conversely, other studies have revealed that the LSA and DSA exhibit minimal to negligible effect on range of motions and functional outcomes [10–12]. Although it has been shown that lateralized RSA offers beneficial outcomes for some ranges of motion, similar associations were not found for between LSA and clinical outcomes. This may be related to the fact that an angle, like for the LSA and DSA, is not giving information on distances. Therefore, a measurement method including a distance ratio would create an index that in theory may be more accurate in predicting clinical outcomes.

The primary objective of this study was to evaluate the validity of the LSA and DSA in estimating implant lateralization and distalization. We hypothesized that the LSA and DSA are not reliable measurements to estimate implant lateralization and distalization. The second aim was to define a new measurement method to better predict lateralization and distalization by using a 3D planning software and virtual range of motion testing and compare it with the existing method.

## Materials and methods

After receiving approval from the institutional review board, shoulder computed tomography (CT) scans of 30 patients with cuff tear arthropathy scheduled to undergo RTSA were evaluated. The study focused only on analyzing anonymous image data from a consecutive case series. Shoulders with severe bone deformity on the glenoid and humeral side were excluded.

The following shoulder characteristics were recorded for all patients: CSA, glenoid inclination (GI), distance from acromion to humeral head (initial distalization), distance from glenoid to greater tubercle (initial lateralization). The CSA was measured by the angle formed between the line connecting the superior and inferior poles of the glenoid and the line connecting the lateral edge of the acromion to the inferior pole of the glenoid according to Bouaicha [13]. 

All scans were analyzed using the Blueprint (Wright, Memphis, TN, USA) software platform. For each patient 24 different RSA configurations were created being composed of 2 base plate types (0 mm and + 6 mm lateralization), 4 different glenospheres (36 mm; 36 mm + 2 mm eccentricity; 42 mm; 42 mm + 4 mm eccentricity), and 3 different tray thicknesses (0 mm, 6 mm and 12 mm). Each model consisted of a Perform Reversed baseplate and an Ascend Flex humeral component with an onlay tray and a neck-shaft-ange of 145°. A total of 720 different configurations were produced. For each configuration, LSA and DSA angles were measured. In accordance with Boutsiadis [7] description the LSA was measured as the angle formed by two tangents originating from the acromion to the greater tuberosity and superior glenoid tuberosity. Likewise, the DSA was measured as the angle between two tangents extending from the superior glenoid to the greater tuberosity and the acromion. The lateralization and distalization arm changes, as computed by the software, were also combined with the initial measurements of lateralization and distalization and then recorded for each configuration separately.

### Lateralization index

**Fig. 1:**
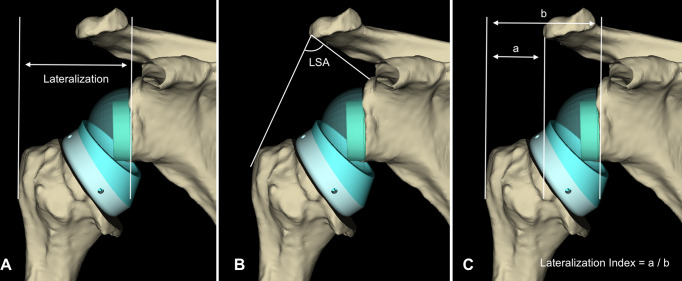
Illustration of lateralization parameters on anteroposterior images. (**A**) Global lateralization is the distance from the glenoid to the lateral border of the greater tuberosity. (**B**) The lateralization shoulder angle (LSA) is the angle formed by two tangents originating from the acromion to the greater tuberosity and superior glenoid tuberosity. (**C**) The lateralization index (LI) is the ratio of the distance from the most lateral aspect of the greater tuberosity to the lateral border of the acromion and the distance from the glenoid plane to the most lateral aspect of the greater tuberosity

To evaluate lateralization three parallel lines were made on anteroposterior images, taking the reference points to be the plane of the glenoid cavity, the lateral border of the greater tuberosity and the lateral border of the acromion. The lateralization index (LI) was defined as the ratio of the distance from the most lateral aspect of the greater tuberosity to the lateral border of the acromion and the distance from the glenoid plane to the most lateral aspect of the greater tuberosity (Fig. 1).

### Distalization index

**Fig. 2:**
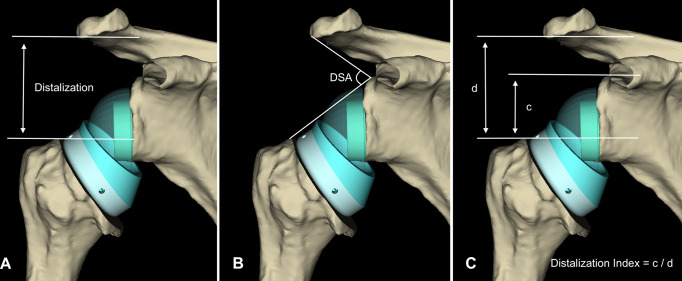
Illustration of distalization parameters on anteroposterior images. (**A**) Global distalization is the distance from the acromion to the most superior border of the greater tuberosity. (**B**) The distalization shoulder angle (DSA) is the angle between two tangents extending from the superior glenoid to the greater tuberosity and the acromion. (**C**) The distalization index (DI) is the ratio between the distance from the most superior aspect of the greater tuberosity to the superior border of the glenoid and the distance from the inferior aspect of the acromion to the most superior aspect of the greater tuberosity

To determine distalization, another three parallel lines were made on anteroposterior images, taking the reference points to be the plane of the inferior border of acromion, the superior border of the glenoid and the most superior aspect of the greater tuberosity. The distalization index (DI) was defined as the ratio between the distance from the most superior aspect of the greater tuberosity to the superior border of the glenoid and the distance from the inferior aspect of the acromion to the most superior aspect of the greater tuberosity (Fig. 2).

To assess intraobserver and interobserver reliability, all the measurements were repeated by the first author (A.E.O.) after a minimum of 2 weeks. These were matched to the findings of the co-author (S.S.).

### Statistical analysis

All statistical analyses were performed using SPSS (version 22.0; IBM). Data were expressed as the mean ± standard deviation. Correlations between angular measurements and the exact distances of lateralization and distalization were analyzed using the Pearson correlation test. Coefficient values (r) between 0.3 and 0.5 indicate a weak correlation, 0.5 and 0.8 indicate a moderate correlation and 0.8 and 1.0 indicate a strong correlation. P value < 0.05 was considered the threshold for significance. An intraclass correlation coefficient of > 0.8 was considered excellent, and a value between 0.5 and 0.8 was considered good. Power analysis showed that our sample size of 720 was sufficient to detect significance with a type I error at 5%, and power of 80%.

## Results

Out of the 30 shoulders, centered glenoid wear was found in 7 patients; posterior wear in 8, posterosuperior wear in 9, and superior glenoid wear in 6 patients. Mean CSA value was 33.6 ± 5.6. Radiological shoulder characteristics are summarized in Table 1. Intraobserver and interobserver reliability values for measurements are shown in Table 2. All measurements showed excellent inter and intraobserver agreement.

**Table 1:** Patient characteristics

Parameters	Mean ± SD
Glenoid Version, °	-13.4 ± 10.6
Glenoid Inclination, °	7.1 ± 10.3
Glenoid Wear Location, n	7 / 8 / 9 / 6
Humeral Inclination, °	130.7 ± 3.2
Humeral Subluxation, %	76.5 ± 14.1
Critical Shoulder Angle, °	33.3 ± 5.6
Reverse Shoulder Angle, °	6.8 ± 4.1

SD: standard deviation; Glenoid Wear Locations: centered / posterior / posterosuperior / superior

**Table 2:** Interclass correlation coefficient for measurements

Measurements	Intra-observer (95% CI)	Inter-observer (95% CI)
Critical Shoulder Angle (CSA), °	0.87 (0.76–0.93)	0.89 (0.80–0.95)
Lateralization Shoulder Angle (LSA), °	0.88 (0.81–0.95)	0.87 (0.81–0.92)
Distalization Shoulder Angle (DSA), °	0.90 (0.81–0.96)	0.91 (0.85–0.97)
Lateralization Index (LI)	0.86 (0.78–0.93)	0.85 (0.75–0.93)
Distalization Index (DI)	0.84 (0.73–0.94)	0.82 (0.74–0.90)
Lateralization, mm	0.86 (0.79–0.89)	0.83 (0.76–0.93)
Distalization, mm	0.82 (0.77–0.88)	0.80 (0.76–0.90)
CI, Confident Interval		

**Fig. 3:**
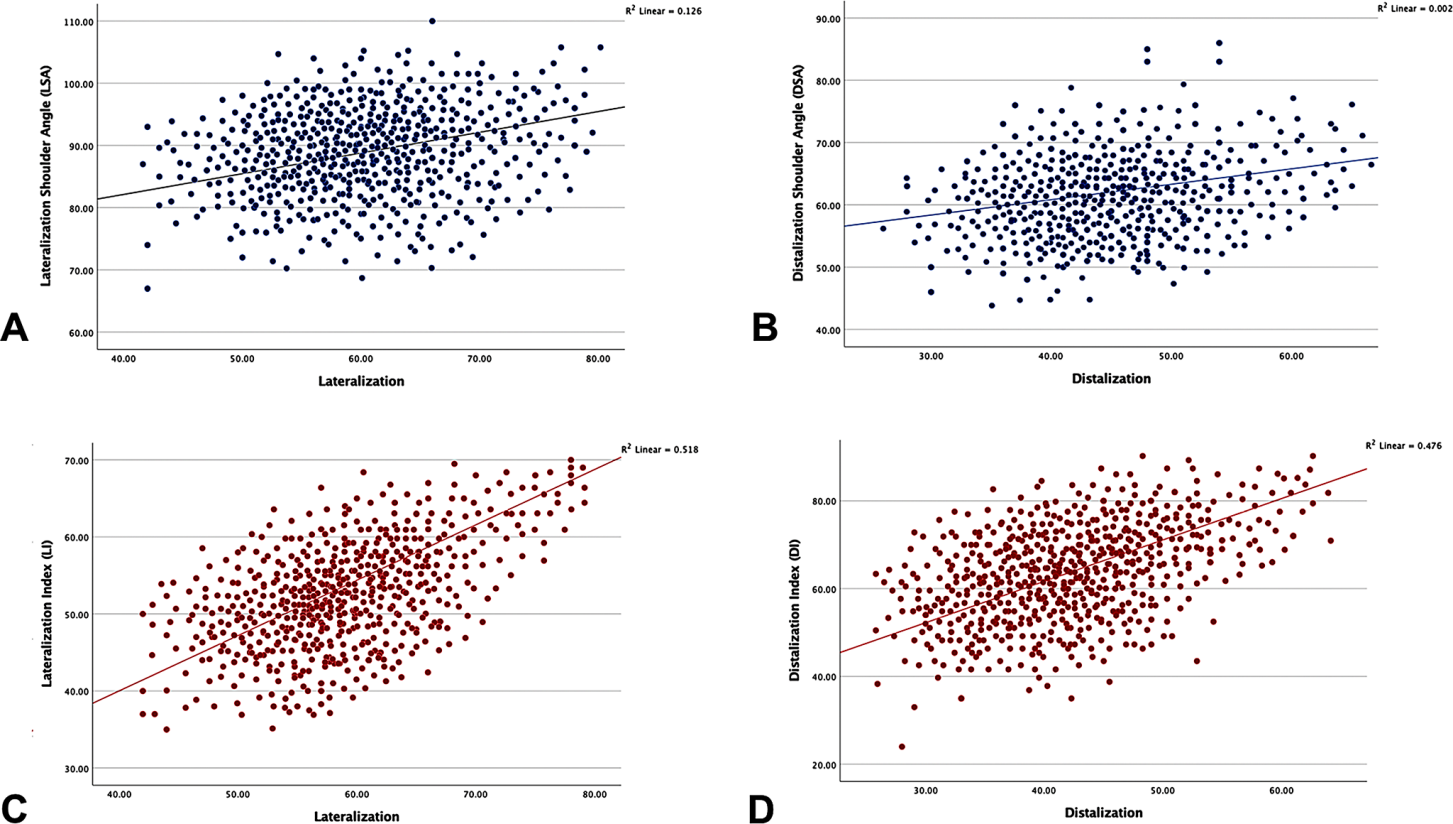
Scatter plots of (**A**) lateralization shoulder angle (LSA), (**B**) distalization shoulder angle (DSA), (**C**) lateralization index (LI) and (**D**) distalization index (DI) and correlations with lateralization and distalization

There were weak but statistically significant correlations between the LSA and lateralization (*r* = 0.36, *p* < 0.01). No significant correlations were found between LSA and LAC. On the other hand, there were moderate correlations between the LI and lateralization (*r* = 0.72, *p* < 0.01) and LAC (*r* = 0.78, *p* < 0.01) (Table 3) (Fig. 3).

**Table 3:** Pearson correlations of LSA and LI

	LSA		LI	
	*r*	*P* Value	*r*	*P* Value
Lateralization	**0.36**	**< 0.01**	**0.72**	**< 0.01**
LAC	0.22	0.065	**0.78**	**< 0.01**
CSA	**0.32**	**< 0.01**	− 0.21	0.135
GI	0.02	0.437	0.09	0.335

CSA: Critical Shoulder Angle, LAC: Lateralization Arm Change, GI: Glenoid Inclination, LI: Lateralization Index, LSA: Lateralization Shoulder Angle. Bold values are statistically significant (*p* < 0.05)

No significant correlations were found between DSA and distalization (*r* = 0.17, *p* = 0.113) and DAC (*r* = 0.06, *p* = 0.214). In contrast, there were moderate correlations between the DI and distalization (*r* = 0.69, *p* < 0.01) and DAC (*r* = 0.62, *p* < 0.01) (Table 4) (Fig. 3).

Both LSA and DSA angles had a moderate correlation with CSA (*r* = 0.54, *r*= -0.74, respectively). No significant correlations were found between GI and LSA, DSA, LI and DI.

**Table 4:** Pearson correlations of DSA and DI

	DSA		DI	
	*r*	*P* Value	*r*	*P* Value
Distalization	0.17	0.113	**0.69**	**< 0.01**
DAC	0.06	0.214	**0.62**	**< 0.01**
CSA	**− 0.54**	**< 0.01**	0.12	0.455
GI	0.09	0.345	0.05	0.539

CSA: Critical Shoulder Angle, DAC: Distalization Arm Change, GI: Glenoid Inclination, DI: Distalization Index, DSA: Distalization Shoulder Angle. Bold values are statistically significant (*p* < 0.05)

## Discussion

The most important finding of this study was that there was a weak correlation between angular radiographic measurements namely LSA and DSA and lateralization and distalization. The new measurement methods (LI and DI) that we propose were more reliable than angular radiographic measurements for estimating lateralization and distalization of the final RSA construct.

Radiographs are easily available both pre- and postoperatively, yet they often lack a calibration object, making length measurements unreliable. Therefore, angle or index measurements on radiographs serve as a straightforward and reliable method for obtaining comparable data on implant positioning. However, there is ongoing debate about the prognostic value of the LSA and DSA on clinical outcomes [7, 11]. While initial findings indicated a significant association between LSA and DSA and improving clinical function, subsequent studies emphasized that these factors have minimal to no impact on predicting clinical outcomes [7, 10]. Studies also highlighted the adequate intraobserver and interobserver reliability of these angular radiographic measurements [12]. In fact, both LSA and DSA measurements are simple and reproducible methods. However, our results showed that these angles were not reliable measurements to estimate implant lateralization and distalization. This could be explained by some geometrical factors that associated measurement method.

**Fig. 4:**
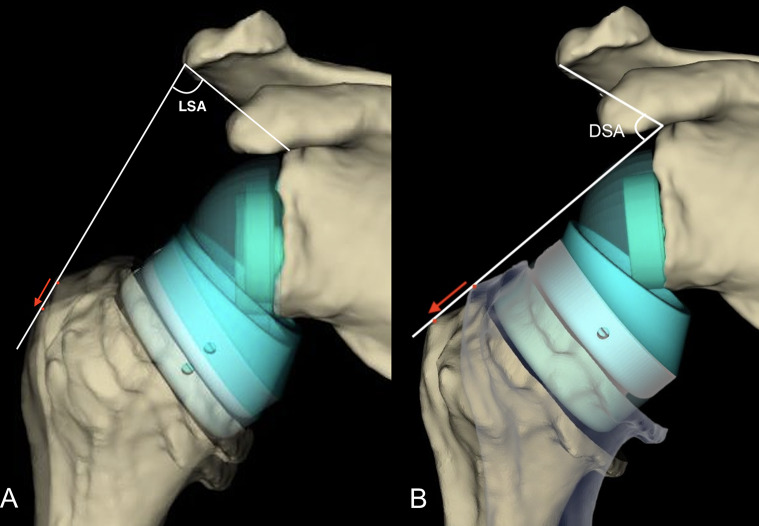
Illustration of two possible scenarios on anteroposterior images. (**A**) Two reverse shoulder arthroplasty (RSA) configurations, one with more lateralized and distalized glenospheres, giving the same lateralization shoulder angle (LSA) angles. (**B**) Two different RSA configurations, one with increased humeral tray thickness, giving the same distalization shoulder angle (DSA) angles

Both LSA and DSA angles are formed by two intersecting lines. In fact, the distance between lines expands when the angle is higher. However, the distance between two lines is not solely determined by the angle between the lines; it also depends on the lengths of the line segments. While the angle between two lines provides information about their relative orientation, the distance between them is a more complex measure that depends on the geometric relationship, the lengths of line segments, and trigonometric functions. Therefore, the lengths of the line segments also affect the distance between two lines. Longer line segments, even if forming the same angle, result in a greater distance between the lines. In this context, it is possible to create an RSA construct that can be more lateralized or distalized without changing the LSA and DSA angles (Fig. 4) (The video illustration is shown in detail in the online Video Supplement).

Another related issue is that the DSA and LSA measurements share some anatomical landmarks with CSA [14]. This means that patient morphological factors may influence angular measurements. However, in an ideal method patient anatomy should have minimal impact on the measurements of distalization and lateralization. In a recent study, Marsalli et al. [14]. showed that DSA had a significant correlation with the CSA. Similarly, our study showed that both LSA and DSA angles had a statistically significant correlation with CSA (*r* = 0.32, *r*= -0.54, respectively). This suggests that even if achieving the same distalization in two different patients, a greater CSA will lead to a decreased DSA measurement.

The new measurement methods that we propose are based on reliable bony landmarks and it is possible to reliably estimate global lateralization and distalization in RSA. Our results showed that both LI and DI measurements had a significant correlation with lateralization and distalization (*r* = 0.72, *r* = 0.66; respectively). Theoretically, LI and DI reflect the amount of lengthening in the remaining cuff muscles and the involvement of deltoid fibers in the overall range of motion [15]. Although some studies reported no significant differences in functional outcomes between lateralized and its nonlateralized group, it has been shown that lateralization is effective in avoiding scapular notching and in retensioning remnant rotator cuff which may increase range of motion [3, 16, 17]. This could be explained by the findings of the study by Imai [18], which reported that the absence of individualized adjustments may impede the lateralization effect. Imai suggested that an 8 mm lateralization has a different effect on remnant muscle tension in 155 and 175 cm tall patients. By the same point of view, effective lateralization and distalization may be better estimated using the LI and DI, rather than using a certain lengths or angles.

In a recent study, Berhouet et al. [19] reported that arm changing position provides useful information to optimize the joint mobility after RSA. The authors emphasized that lateralization and distalization arm change were significantly associated with better range of motion. While our findings showed no significant correlation between arm changing position and LSA and DSA, there was a strong correlation with LI and DI (*r* = 0.78, *r* = 0.62; respectively). Thus, these measurements may also have the potential to predict the functional outcomes after RSA.

The principal finding drawn from this study is that LSA and DSA are unreliable for estimating implant lateralization and distalization. Thus, we have introduced two alternative measurement methods, LI and DI. These measurements are conducted on postoperative true anteroposterior radiographs, which are obtainable in clinical settings. However, a critical aspect of this method is obtaining accurate radiographs. Inferences should not be drawn from measurements taken on incorrect images. Moreover, utilizing a two-length ratio as a radiological index can be difficult but it is more reliable than using angle or length measurements to obtain comparable data on implant positioning.

### Limitations

The limitations of this study are typical of computer-based simulations. We created 720 different configurations from 30 shoulders to analyze the effectiveness of measurement methods to estimate implant lateralization and distalization. We introduced two new measurement methods, however their value as prognostic factors for clinical outcomes in patients after RSA is still unknown. Future studies are needed to validate the clinical significance of these methods. Moreover, scapular parameters including acromial length and height may also influence LI and DI measurements similarly to LSA and DSA measurements, thus lead to variability among patients. Nevertheless, our results showed that LI and DI are more reliable methods.

## Conclusion

LI and DI are more reliable methods to estimate implant lateralization and distalization compared to angular radiographic measurements. However, the prognostic significance in predicting clinical outcomes after RSA remains unknown.

## Electronic supplementary material

Below is the link to the electronic supplementary material.

Supplementary Material 1

Supplementary Material 2

Supplementary Material 3
