# Supplementary material for: The lateralization and distalization index is more reliable than angular radiographic measurements in reverse shoulder arthroplasty
Source: Arch Orthop Trauma Surg. 2024 Jul 15;144(8):3247–53. doi: 10.1007/s00402-024-05448-6 (PMC11417055; doi:10.1007/s00402-024-05448-6)
Supplement: Supplementary file 3 — Supplementary Material 3 [file 402_2024_5448_MOESM3_ESM.docx]

**Legend of Videos:**

**Video 1.** In the video demonstration, the RSA construct appeared to exhibit increased lateralization and distalization, yet the LSA angles remained unchanged.

**Video 2.** The video demonstration illustrated a RSA construct that was notably more distalized using a more thick tray, however the DSA angles remained constant.
